# A Review of Zinc-L-Carnosine and Its Positive Effects on Oral Mucositis, Taste Disorders, and Gastrointestinal Disorders

**DOI:** 10.3390/nu12030665

**Published:** 2020-02-29

**Authors:** Susan Hewlings, Douglas Kalman

**Affiliations:** 1 Central Michigan University, Department of Nutrition and Dietetics Mount Pleasant, MI 48859, USA; 2 College of Healthcare Sciences, Nova Southeastern University, Fort Lauderdale 33314, USA; dkalman@nova.edu

**Keywords:** zinc, carnosine, oral mucositis, taste disorders

## Abstract

Zinc-L-carnosine (ZnC), also called polaprezinc known as PepZin GI™, is a chelated compound that contains L-carnosine and zinc. It is a relatively new molecule and has been associated with multiple health benefits. There are several studies that support ZnC’s benefits in restoring the gastric lining, healing other parts of the gastrointestinal (GI) tract, improving taste disorders, improving GI disorders, and enhancing skin and liver. Oral mucositis is a common complication of cytotoxic radiotherapy and/or chemotherapy. It occurs in almost every person with head and neck cancer who receive radiotherapy. It is often overlooked because it is not considered life threatening. However, mucositis often leads to a decreased quality of life and cessation of treatment, ultimately decreasing positive outcomes. Therefore, solutions to address it should be considered. The primary mechanisms of action are thought to be localized and related to ZnC’s anti-inflammatory and antioxidant functions. Therefore, the purpose of this review is to discuss the research related to ZnC and to explore its benefits, especially in the management of conditions related to damaged epithelial cells, such as oral mucositis. Evidence supports the safety and efficacy of ZnC for the maintenance, prevention, and treatment of the mucosal lining and other epithelial tissues. The research supports its use for gastric ulcers (approved in Japan) and conditions of the upper GI and suggests other applications, particularly for oral mucositis.

## 1. Introduction

Zinc-L-carnosine (ZnC), also called polaprezinc, is a chelated compound that contains L-carnosine and zinc [1]. Zinc is a required mineral found in meat, eggs, shellfish, cheese, legumes, and tofu. Zinc is an essential mineral that is a part of many enzymes that are critical in cell proliferation during cell repair, especially in epithelial and epidermal cells [2]. Therefore, it is required for wound healing of the skin, connective tissue, and intestinal lining, particularly epithelial tissue. Zinc deficiency, whether from dietary, hereditary, or other causes, leads to pathological conditions, such as growth retardation, skin symptoms, and taste disorders [3].

L-carnosine is also part of ZnC. β-Alanyl-l-histidine is a dipeptide and a chelator of metal ions. It is found in the muscles of vertebrates, and therefore dietary meats. It has been shown to play a protective role in wound healing, immune function, diabetes, and loss of vision, and this is thought to be due to its role as a buffer and as an antioxidant [1,4].

The combination or chelation of the zinc and carnosine that results in ZnC is said to have superior health benefits compared to either alone as carnosine enhances the absorption of zinc because of its solubility and perhaps because it delivers zinc to the tissues in a delayed/extended release manner [1]. In the US, ZnC is licensed as a dietary zinc supplement and a possible adjunctive agent to promote the restoration of a healthy gastric lining in people with peptic ulcers [5]. Known as PepZin GI™ (XSTO Solutions), this is the only form of ZnC that has been reviewed for safety and human use by the United States Food and Drug Administration (FDA) it was granted “new dietary ingredient” status in 2002 [6]. While there are several studies reporting its efficacy in restoring the gastric lining [7], there is evidence to suggest it restores tissue in other parts of the GI tract as well. For example, there is research to support its role in the management of taste disorders [8,9,10], GI disorders [11,12,13,14,15,16,17], skin [18,19], liver [20,21], and oral mucositis resulting from chemo and or radiotherapy [22,23,24,25]. This makes sense considering that these soft wet tissues are all lined with epithelial tissues, and zinc plays a key role in maintaining the health and repair of epithelial tissue [3].

Oral mucositis is a common complication of cytotoxic radiotherapy and/or chemotherapy, affecting 75% of high-risk patients [26]. It occurs in almost every person with head and neck cancer who receive radiotherapy [27,28]. It is associated with severe pain, odynophagia, dysgeusia, malnutrition, and dehydration, which severely impairs patients’ quality of life [29,30]. While it is a very serious and common side effect of radiation and/or chemotherapy, affecting hospital stays and overall outcomes, there are few options for treatment, making alternative treatments with established safety and efficacy warranted [26]. Therefore, the purpose of this review was to discuss the research related to one potential treatment, ZnC, and to explore its benefits, especially in the management of conditions related to damaged epithelial cells.

## 2. Mechanism of Action of ZnC

Several studies have suggested that ZnC reduces gastric lesions related to stomach ulcers and accelerates the healing process in animal models [31,32,33,34]. The primary mechanisms of action are thought to be related to its anti-inflammatory and antioxidant functions [35]. For example, in an ethano-induced rat model of gastric injury, inflammatory cytokines, such as interleukin-1β, interleukin-8, interleukin-6, and tumor necrosis factor, decreased in a dose-dependent manner in the group receiving ZnC when compared to the control group [31]. It has been reported that NF-kB (Nuclear Factor kappa-light-chain-enhancer of activated B cells), one of the main transcriptional factors that regulates the expression of several genes involved in inflammation and immune responses, is inhibited by ZnC supplementation. Several in vitro and animal models support these findings [5,32,36]. Additionally, antioxidant markers, such as superoxide dismutase-1, superoxide dismutase-2, hemeoxygenase-1, peroxiredoxin-1, and peroxiredoxin-V, were increased in the group receiving ZnC compared to the treatment group in the rat model [31]. Other studies have supported these findings [32,37,38]. Additionally, growth factors, such as vascular endothelial growth factor, nerve growth factor, and platelet-derived growth factor, were significantly increased in the group receiving ZnC [31,36]. Other mechanisms of action, whereby ZnC may exert a beneficial effect, are via heat shock proteins, which have been shown to be cytoprotective. It has been reported that ZnC supplementation increases heat shock proteins in rodent models [31,39,40]. The antioxidant function of ZnC is supported by in vitro studies [41], as well as human intervention studies. For example, 12-week placebo-controlled study of subjects aged 65 to 85 years with low plasma zinc status (lower than 0.77 mg/l (11.77 mM) (n D 90) were randomized to receive a ZnC supplement or placebo. The ZnC-supplemented group was given a ZnC tablet containing 86.9 mg of ZnC (equivalent to 20 mg of zinc) daily. The ZnC-supplemented group was found to have higher ferric reducing ability of plasma and erythrocyte superoxide dismutase (eSOD) activity as compared to baseline; however, only eSOD activity was significantly higher than the placebo group [42,43]. 

An invitro study examined the effect of ZnC on cellular activity involved in the early (pro-migratory, restitutive) and later (pro-proliferative) stages of healing. Pro-migratory (restitutive) activity of ZnC was assessed using the human colonic carcinoma cell line HT-29, in a wounded monolayer assay system with serial photomicrographs. Effects on cell proliferation were assessed using [3H] thymidine incorporation assays using the human intestinal cell lines IEC-6 and HT-29 and also the rat intestinal epithelial cell line RIE-1. The addition of ZnC to HT29 cells caused a pro-migratory activity in a dose-dependent manner, with a maximum effect seen at 100μM, causing a significant (*p* < 0.01) approximate doubling of the rate of wound closure. Addition of ZnC increased the proliferation of HT29 and RIE-1 cells in a typical a bell-shaped dose–response fashion. Peak stimulation (about 160% above baseline levels) was seen at 34 μM in both HT29 and RIE-1 cells (*p* < 0.01 vs. control). No pro-stimulatory effect of ZnC was seen in IEC-6 cells. The authors concluded that ZnC, at concentrations similar to those likely to be found in the gut lumen, is capable of stimulating both the early and later stages of gut repair when tested using in vitro models of intestinal injury [44]. While this study was conducted in colonic cells, it can be applied to other epithelial cells. 

## 3. Oral Mucositis

Oral mucositis is a common complication of cytotoxic radiotherapy and/or chemotherapy. It occurs in almost every person with head and neck cancer who receive radiotherapy [26,27,28]. It is associated with severe pain, odynophagia, dysgeusia, malnutrition, and dehydration, which severely impairs patients’ quality of life [29,30]. It can be the cause of an extended hospital stay [45] and may even be the cause of early cessation of therapy [46]. Therefore, strategies to prevent mucositis can potentially improve outcomes and decrease hospital stays. A prospective study was conducted to assess the feasibility and efficacy of administering ZnC in the form of an oral rinse for radiation-induced mucositis in head and neck cancer patients while they were undergoing radiotherapy. The rinse had a concentration of 37 mg/dl ZnC, it was given to patients 4 times per day in the hospital, and the rinse was kept in the mouth for 1 min. This dose equated to the recommended ZnC dose of 150 mg/day. Of the patients who received the rinse, 29% developed grade 3 mucositis based on the mucosal findings and 39.3% based on the self-report symptoms. Of those who did not receive the rinse, 40% developed grade 3 based on the mucosal findings and 60.7% based on the self-report symptoms. The oral rinse was well tolerated, and the authors concluded that it was a promising treatment for mucositis [22].

Recently, it was reported that oral administration of ZnC suspension in sodium alginate (P-AG) is effective for the prevention of oral mucositis associated with radiotherapy for head and neck cancer [23], high-dose chemotherapy, and radiotherapy before hematopoietic stem cell transplantation [24]. The exact benefits of the supplementation were clarified in a follow-up assessment of medical charts and it was concluded that P-AG reduced the irradiation period and the time to discharge after the completion of radiotherapy by preventing oral mucositis in patients with head and neck cancer [46]. Similarly, a study using a lozenge preparation of 18.75 mg ZnC demonstrated a significant reduction of 13% in the grade of oral mucositis as well as a 13% reduction for pain medication in patients receiving high-dose chemotherapy for hematopoietic stem cell transplantation [47].

ZnC has been shown to prevent oral mucositis for patients with other types of malignancies receiving radiochemotherapy. A study of 36 patients with hematological malignancy receiving high-dose chemotherapy and radiotherapy followed by hematopoietic stem cell transplantation (HSCT) were given a mouth rinse containing ZnC (0.5g suspended in 20mL of 5% sodium alginate), P-AG 4 times per day for 2 min of rinsing, and then swallowed for one month after transplantation. The comparison group were treated with azulene gargle for one month after transplantation. The ZnC rinse reduced the incidence of moderate to severe oral mucositis as compared to the control group treated with azulene gargle (20% versus 82% for grade ≥2, *p* < 0.01; 0% versus 45% for grade ≥3, *p* < 0.01). Pain associated with oral mucositis was also significantly (*p* = 0.004) relieved, resulting in a reduction in the use of analgesic agents (28% versus 73%, *p* = 0.025). The incidence of xerostomia and taste disturbance tended to be lowered but not significantly by P-AG. On the other hand, the rinse had no influence on the incidence of other adverse events, tumor remission rate, or the survival rate. The authors concluded that ZnC rinse was found to be highly effective in preventing oral mucositis induced not only by radiochemotherapy for head and neck cancer but also by high-dose chemotherapy and radiotherapy followed by HSCT [24].

Ishihama et al. reported that a ZnC mouth rinse was effective for improving oral mucosal injury in 423 patients who were experiencing symptoms of oral mucosal injury as a result of cancer treatment. Effects of the ZnC rinse were examined according to the cancer treatment method: The stomatitis prevention success rate, symptom improvement rate, pain prevention success rate, and symptom improvement rate were 68.5%, 84.4%, 75.4%, and 76.7%, respectively, for chemotherapy (*n* = 280); 32.7%, 64.5%, 45.5%, and 73.5% for chemoradiation therapy (*n* = 95); and 29.6%, 60.0%, 40.7%, and 68.6% for radiotherapy alone [25].

In addition to effectively preventing mucositis, ZnC has been shown to prevent esophagitis occurring as a complication of chemoradiotherapy. Patients with non-small cell lung cancer who received a weekly administration of carboplatin and paclitaxel combination chemotherapy with a concurrent thoracic radiotherapy were evaluated. Patients (*n* = 19) who received ZnC were compared to those who did not (*n* = 19). Patients who received an oral mixture of 60 mL of sodium alginate solution and 150 mg of ZnC 3 times per day before meals were compared to those who did not receive ZnC but did receive 20mL of sodium alginate solution 3 times per day orally before each meal as well as aluminum-magnesium hydroxide gel throughout the radiation therapy. The development of grade ≥2 radiation esophagitis was significantly inhibited by ZnC at the dose of 37.5 or 75 mg twice a day (HR, 0.397; 95% CI, 0.160–0.990; *p* = 0.047). The median onset was 21.0 days for the control group, while the value did not reach significance in the experimental group. Supplementation of ZnC reduced the incidence of grade >2 esophagitis at the lower dose of radiation but not the higher dose, suggesting that ZnC causes a delay in the onset of grade ≥2 esophagitis [11].

## 4. Uses in Taste Disorders

Taste disorders are often associated with oral mucositis and other side-effects of chemo and radiation therapy. Taste disorders are common worldwide yet are poorly studied, perhaps because they are not considered serious or life threatening. However, they affect as many as 90% of patients receiving radiotherapy for head and neck cancers [48], impact the quality of life, and can indirectly influence the outcomes of more serious conditions by decreasing nutritional intake [49]. Enzymes that require zinc are contained in the taste buds and play an important role in the function of taste [3]. Several studies have demonstrated that zinc supplementation improves taste in those with taste disorders not associated with cancer [50,51,52]. The effect of ZnC on taste disorders was assessed in a randomized, double-blind, placebo-controlled trial. In total, 107 subjects suffering from taste disorders not associated with cancer were assigned to receive placebo, 75 mg, 150 mg, or 300 mg of ZnC orally for 12 weeks. Taste perception was assessed using the paper filter disk (PFD) and a subjective questionnaire and serum zinc were measured before and after supplementation. Subjects receiving the 300-mg dose demonstrated a significant improvement compared to the placebo group. Subjective report symptoms improved in the groups receiving the 150-mg and 300-mg doses. Serum zinc increased in a dose-dependent manner, with the group that received the highest dose of ZnC demonstrating a statistically significant increase from baseline. No serious adverse events were reported [8]. 

Another study was conducted to assess the effect of 150 mg of ZnC supplementation in 40 patients complaining of taste impairment. The patients were screened for serum zinc levels and divided into two groups. The zinc-deficient taste disorder group were those with serum zinc of less than 63 µg/dl with no history of other disorders; those with values higher than that were placed in the idiopathic group. Both groups received 150 mg of ZnC for an average of 17.7 weeks. Subjective symptoms were measured via the visual analogue scale (VAS). There were no statistically significant differences in subjective symptoms between the groups at baseline. Interestingly, supplementation improved symptoms significantly in both groups. There were no correlations between the VAS scores and serum zinc levels at the end of the study, suggesting that zinc deficiency or impairment may be present even when serum zinc levels are not below recommended levels [9].

In a single-center retrospective study, subjects who showed grade 2 taste disorders as a result of chemotherapy were give 150 mg of ZnC 2x/day until the symptoms disappeared. A comparison group were given an azulene gargle solution. The median time to recovery was significantly shorter in the group receiving the ZnC compared to the comparison group (63 days compared to 112 days, hazard ratio (HR), 1.778; 95%CI = 1.275.2.280; *p* = 0.019). A multivariate regression analysis revealed that pancreatic cancer and use of fluoropyrimidines increased the risk of developing grade 2 taste disorder. This is most likely because secretions from the pancreas are involved with zinc absorption. Additionally, subjects with pancreatic cancer did not respond as well as those without pancreatic cancer to the oral supplementation [10].

## 5. Gut Mucosal Integrity

ZnC is perhaps best known for its approved use in Japan for the management of stomach ulcers. In a randomized, controlled, double-blind study, 258 subjects with confirmed stomach ulcers were randomly assigned to receive 150 mg ZnC per day, a placebo, 800 mg of cetraxate hydrochloride (a known mucosal protection agent), or its placebo for 8 weeks. Endoscopy was done before and after treatment and subjective measures of symptoms were collected. Symptoms were 61% better in the marked improvement category in the ZnC group and 61.5% in the cetraxate group at 4 weeks. At 8 weeks, the ZnC group increased to 75% markedly improved compared to 72% for the cetraxate group. The endoscopic cure rate was 26.3% in the ZnC group and 16.2% in the cetraxate group at 4 weeks and 60.4% in the ZnC group and 46.2% in the cetraxate group at 8 weeks. This suggests that ZnC can provide superior relief of symptoms and improvement in gastric ulcers compared to a known mucosal protection agent [12]. Another study by the same group using 50, 75, or 100mg twice daily showed improvement in symptoms and the endoscopic healing rate at all three doses [53]. Other human clinical trials support these results at doses of 50, 75, and 100 mg twice a day [13,14,15,16,17].

In an ethanol-induced gastro-injury rat model, ZnC treatment decreased the ulcer index of the rat stomach and showed a significant ulcer-healing effect similar to the gastric mucoprotective agent rebamipide [31]. Similarly, a significant ulcer healing effect was reported with ZnC supplementation compared with the placebo treatment group in an aspirin-induced gastroduodenal injury animal model [32,33]. In addition, a study using an acetic acid-induced rat model reported that the ZnC treatment group showed a significant antiulcer effect and healing action compared to the control [34]. These results are most likely a result of the anti-inflammatory and antioxidant functions of ZnC [35]. This function of ZnC helps to explain the many other benefits it can exert throughout the GI tract.

It has been reported that ZnC stimulated several aspects of gut mucosal integrity. In vitro studies using pro-migratory (wounded bilayer) and proliferation ([(3)H]-thymidine incorporation) assays of human colonic (HT29), rat intestinal epithelial (RIE), and canine kidney epithelial cells showed that ZnC stimulated cell migration and proliferation and reduced the amount of gastric and small intestinal injury in rats and mice. In vivo studies used a rat model of gastric damage (indomethacin/restraint) and a mouse model of small-intestinal (indomethacin) damage. Oral ZnC decreased gastric (75% reduction at 5 mg/mL) and small-intestinal injury (50% reduction in villus shortening at 40 mg/mL; both *p* < 0.01). In a cross over study of 10 healthy human subjects comparing changes in gut permeability (lactulose/rhamnose ratios) before and after 5 days of indomethacin treatment (50 mg three times a day) with ZnC (37.5 mg twice daily) or placebo, it prevented the rise in gut permeability caused by indomethacin [7].

In an in vitro model, the effect of ZnC on cellular activity in the early (pro-migratory) and later (pro-proliferative) stages of healing were assessed using a human colonic carcinoma cell line in a wounded monolayer assay system with serial photomicrographs. Effects on cell proliferation were assessed using [3H] thymidine incorporation assays using the human intestinal cell lines IEC-6 and HT-29 and also the rat intestinal epithelial cell line RIE-1. Indices of early and late-stage gut repair were stimulated. Addition of the ZnC to the cells caused a dose-dependent increase in pro-migratory activity, leading to a doubling of the rate of wound closure. Proliferation was also increased in a dose-dependent bell-shaped pattern [44]. Similarly, it was shown to protect rat small intestinal epithelial cells from acetyl salicylic acid-induced apoptosis damage by increasing heat shock proteins without negatively impacting the cell [54].

Drug-induced enterocolitis is caused by a number of morphological and functional changes of the small and large intestine resulting from short or long-term exposure to medications. This is a very common side-effect of many medications. GI disorders and hepatoxicity are the most frequent adverse drug reactions that cause licensed drugs to be withdrawn from the market. GI events, such as diarrhea and constipation, are the most common GI-associated adverse drug events and are often associated with non-steroidal anti-inflammatory drugs (NSAIDs) and antibiotic use [55]. In fact, as many as 70% of NSAID users have intestinal mucosal injuries, such as lesions, erosions, and even ulcers [56]. It has been suggested that the anti-inflammatory and antioxidant properties of ZnC as well as its ability to upregulate heat shock proteins may prevent mucosal injury by NSAIDs [55]. It has been reported that nuclear factor kappa-light-chain-enhancer of activated B cells, a pro-inflammatory molecule, was suppressed for 6 h after administration of ZnC in rats [57].

A randomized, parallel-group, open-label, controlled, prospective multicenter study was conducted to assess the efficacy and safety of ZnC combined with triple therapy (omeprazole 20 mg, amoxicillin 1 g, and clarithromycin 500 mg) and compared to triple therapy alone as therapy to eradicate *Helicobacter pylori*. Subjects (*n* = 303) were randomly assigned to receive triple therapy plus 75 mg of ZnC twice a day, triple therapy plus 150 mg of ZnC twice a day, or triple therapy alone. Intention-to-treat (ITT) analysis showed that the rate of *H. pylori* eradication was significantly higher for arms A (77.0%) and B (75.9%) compared to arm C (58.6%) *p* < 0.01), whereas there was no difference between arms A and B (*p* = 0.90). Per-protocol analysis showed that the rate of *H. pylori* eradication was significantly higher for arms A (81.1%) and B (83.3%) compared to arm C (61.4%) (*p* < 0.01), whereas there was no significant difference between arms A and B (*p* = 0.62). All three groups reported significant symptom improvement at 7, 14, and 28 days after treatment, compared to baseline (*p* < 0.0001). The adverse event rate for arm B (5.1%) was higher than for arms A (2.8%) (*p* = 0.04) and C (1.9%) (*p* = 0.02). There were no serious adverse events in any group. The authors concluded that ZnC is a safe and well-tolerated adjunct to triple therapy to eradicate *Helicobacter pylori* [58]. These results are supported by those of Ko et al., who reported rats with ulcers given 30 and 60 mg/kg of ZnC for 3 days experienced a significant reduction in the gastric ulcer area in a dose-dependent manner with an associated increase in xanthine oxidase and myeloperoxidase activities as well as malondialdehyde in the ulcerated mucosa. The mucosal glutathione was also restored. ZnC also caused the overexpression of basic fibroblast growth factor, vascular endothelial growth factor, and ornithine decarboxylase. ZnC consistently downregulated the protein expression of tumor necrosis factor-α, interleukin-1β, macrophage inflammatory protein-2, and cytokine-induced neutrophil chemoattractant-2α that was activated in the ulcerated tissues. The authors concluded that ZnC promotes a healing effect through its antioxidant effects [36]. While Handa et al. suggested that ZnC may inhibit *H. pylori*-induced polymorphonuclear leukocyte-mediated gastric inflammation by reducing leukocyte CD11b/CD18 integrin expression and the production of proinflammatory cytokine interleukin-8 in gastric epithelial cells [59].

## 6. Pressure Ulcers

Pressure ulcers (PUs) are a common and costly issue in all levels of healthcare, especially for bed-ridden patients, and can be very costly and can decrease outcomes regardless of primary diagnosis. PU is defined as “localized injury to the skin and/or underlying tissue usually over a bony prominence, as a result of pressure, or pressure in combination with shear” [60]. The guidelines established by the National Pressure Ulcer Advisory Panel and the European Pressure Ulcer Advisory Panel recommend that “individuals with PUs are provided sufficient calories of 30-35 kcal/kg body weight, adequate protein of 1.25–1.5 g/kg body weight, and, if deficiencies are present, adequate vitamins and minerals” [60] Despite efforts to follow the recommendations and to explore different protein levels, antioxidants, arginine, zinc, and other nutrients, a recent systematic review concluded that there is no clear benefit of nutritional interventions reported at the time of its publication in 2014 [61]. However, it has been reported that there is evidence to support the use of ZnC in the treatment of PUs [18,19]. In a randomized control trial, 42 patients with stage II to stage IV PU were assigned to one of three groups, control(*n* = 14), ZnC 75mg (58mg carnosine and 17mg zinc) orally (*n* = 10), or carnosine 58mg orally only (*n* = 18) for 4 weeks. All other care was the same for the three groups. The rate of pressure ulcer healing, assessed by the mean weekly improvement in the Pressure Ulcer Scale for Healing (PUSH) score, was significantly greater in the carnosine (1.6 ± 0.2, *p* = 0.02) and ZnC groups (1.8 ± 0.2, *p* = 0.009) than in the control group (0.8 ± 0.2). The difference between the carnosine and ZnC groups was not significant (*p* = 0.73) [18]. A second case series study with no control arm from the same group but using comparative data from the earlier study was done over 8 weeks and 19 subjects received 150 mg/day ZnC (116mg carnosine and 34 mg zinc). The PUSH score improved significantly from 8.1 [95% CI, 6.0–10.3] at baseline to −1.4 [−4.0 to 1.1] after 8 weeks (*p* < 0.001). Differences from baseline were significant after 1 week (*p* < 0.05). The mean weekly improvement in PUSH score was 2.0. Eleven patients healed within 8 weeks and none dropped out. Serum zinc levels increased significantly (*p* < 0.001). The authors concluded that the data suggest that ZnC may be effective and well-tolerated in an 8-week treatment of PUs. The study also showed a significant decrease in copper levels, which they suggest should be monitored in future studies. While promising, the results are preliminary and warrant future studies [19].

## 7. Liver

It has been reported that patients with chronic liver disease show impaired trace element metabolism. Specifically, high levels of iron and copper, and low levels of zinc, selenium, phosphorus, calcium, and magnesium [62]. Because of its antioxidant and anti-inflammatory effects, it has been hypothesized that supplemental zinc would be beneficial as an adjunct to treatments for chronic hepatitis C. It has been reported that zinc supplementation enhances the response to interferon therapy in patients with intractable chronic hepatitis C [63]. 

A significant decrease of serum transaminase activities (ALT) was observed in 12 chronic hepatitis C patients who received daily supplementation of 150 mg of ZnC for 48 weeks during combination therapy comprising PEG-IFN-2b plus ribavirin compared to controls (*n* = 12). All patients received 300 mg of vitamin E and 600 mg of vitamin C. Supplementation with ZnC decreased plasma thiobarbituric acid reactive substances (TBARS) concentration and prevented the decrease in erythrocyte polyunsaturated fatty acid (PUFA) levels. The authors hypothesized that the ZnC exerted its benefit via its antioxidant properties and that the PUFA levels were evidence of a decrease in lipid peroxidation. They suggested that since zinc is absorbed in the small intestine and transported to the liver via the portal vein, hepatocytes may be exposed to higher levels of zinc than other tissues, especially during supplementation. The authors concluded that ZnC may offer antioxidant protection for chronic hepatitis patients undergoing treatment [20]. 

A study by Himoto et al. examined the effects of zinc treatment on inflammatory activities and fibrosis in the liver of patients with hepatitis C virus (HCV) and chronic liver disease (CLD). Fourteen patients with HCV-related chronic hepatitis and liver cirrhosis, defined by a persistent elevation of serum aspartate aminotransferase (AST) and/or ALT at more than twice the normal upper limits for at least 6 months, participated in the study. Subjects received 75 mg ZnC 3 x/day for 6 months in addition to their prescribed medications. Peripheral blood cell counts, serum liver-related biochemical parameters that reflect hepatic reserve and inflammatory activity, genotypes, and loads of HCV-RNA, serological markers for liver fibrosis including type IV collagen 7S and hyaluronic acid, and serum levels of trace elements, such as zinc and copper, iron, and ferritin, were examined before and after supplementation. Serum zinc concentrations were positively correlated with hepatic reserve before zinc supplementation. A significant increase in serum zinc levels was observed after supplementation. Supplementation significantly decreased serum aminotransferase levels, and alkaline phosphatase levels were significant. Serum ferritin levels were significantly decreased. The reduction rate of ALT levels was positively correlated with that of ferritin. There was a tendency toward a decrease in serum type IV collagen 7S levels after supplementation. However, peripheral blood cell counts, other liver function tests, or HCV-RNA loads were not affected. At this level of supplementation, copper levels were not affected while serum ferritin levels were decreased. The authors suggested that supplementation with ZnC leads to a decrease in liver inflammation in patients with HCV-related CLD through its antioxidant effects, thereby preventing iron-induced free radical activity [21].

Nishida et al. reported that ZnC and zinc sulfate alone but not L-carnosine alone increased HSP70 and prevented acetaminophen toxicity in mouse primary-cultured hepatocytes. Cell death and lipid peroxidation were suppressed as well. The results suggest a cytoprotective effect of ZnC, especially related to the zinc component in hepatocytes experiencing acetaminophen toxicity [64].

## 8. Safety

A potential concern with zinc administration is possible induction of copper deficiency because high doses of zinc are known to inhibit copper absorption. However, a typical dose of ZnC is 22% zinc (and 78% L-carnosine), which would typically deliver approximately 15 mg (or 15–16 mg) of zinc, which should not be a concern. In addition, ZnC has a long-established safety profile based on long-term use in humans with no adverse events reported as well as several pre-clinical and human clinical studies [65,66].

## 9. Conclusions

In conclusion, evidence supports the safety and efficacy of ZnC for the maintenance, prevention, and treatment of the mucosal lining and other epithelial tissues. This supports its allowed use as a dietary zinc supplement in the US and for radiation therapy and gastric ulcers and suggests other applications, particularly for oral mucositis experienced in cancer patients undergoing chemoradiation treatment and for taste disorders. The results reported regarding efficacy are further supported by ZnC’s anti-inflammatory and antioxidant mechanisms of action. Further randomized controlled studies in humans are warranted. 
